# Global, regional, and national trends and burden of diabetes mellitus type 2 among youth from 1990 to 2021: an analysis from the global burden of disease study 2021

**DOI:** 10.3389/fendo.2025.1626225

**Published:** 2025-10-22

**Authors:** Ling Deng, Shuihua Lu, Jianfeng Zeng, Houming Liu

**Affiliations:** 1 School of Public Health, Shenzhen University Medical School, Shenzhen University, Shenzhen, Guangdong, China; 2 Shenzhen Third People’s Hospital, National Clinical Research Center for Infectious Diseases, Shenzhen, Guangdong, China

**Keywords:** youth, T2DM, GBD 2021, incidence, prevalence, disability-adjusted life years

## Abstract

**Background:**

Although studies have looked at type 2 diabetes mellitus (T2DM) in adults, global studies on youth with T2DM are relatively scarce. Understanding the global, regional, and national trends and burden of T2DM in this special population is critical to developing effective preventive control measures and strategies. Therefore, this study aims to shed light on the specific challenges facing different populations and regions to ultimately guide global action.

**Methods:**

Based on the Global Burden of Diseases, Injuries, and Risk Factors Study 2021, the incidence, prevalence, and Disability-Adjusted Life Years (DALYs) of youth with T2DM aged 15–24 years from 1990 to 2021 were extracted and analyzed at global, regional, and national levels. Point estimates with 95% uncertainty intervals (UIs) were used to calculate the average annual percentage changes (AAPCs) of incidence, prevalence, and DALYs. Subsequently, trends were thoroughly analyzed at the global, regional, and national levels, and the global trends were analyzed in detail by factors like age, sex, and social development index.

**Results:**

The global incidence, prevalence, and DALYs of T2DM in youth increased to varying degrees from 1990 to 2021. With an AAPC of 2.62 (95% CI: 2.42 - 2.81), the global incidence of T2DM increased from 56.0 per 100–000 population in 1990 to 123.9 per 100–000 population in 2021. The incidence of increase in males (AAPC 2.68, 95% CI: 2.47 - 2.89) was higher than in females (AAPC 2.51, 95% CI: 2.35 - 2.66). The incidence increased with increasing age, but the largest increase was found in youth aged 15–19 years (AAPC 2.72, 95% CI: 2.47 - 2.96). High SDI areas saw the greatest increase in incident rates (AAPC 3.48, 95% UI: 3.43 - 3.52) compared with other areas.

**Conclusion:**

The increasing global incidence, prevalence, and DALYs of T2DM in youth presented a large burden to public health over the past thirty years, while the trends and burden vary by region, nation, gender, age, and level of development. Our study highlights the significance of developing targeted public health policies and strategies to respond to the heterogeneity among youth with T2DM.

## Introduction

Diabetes mellitus comprises a spectrum of chronic metabolic disorders defined by hyperglycemia. This condition arises from a defect in insulin secretion, a defect in insulin action (insulin resistance), or most commonly, a combination of these two pathophysiological disturbances (1). According to the etiological evidence of the World Health Organization (WHO), diabetes mellitus can be divided into four types: type 1 diabetes mellitus (T1DM), type 2 diabetes mellitus (T2DM), other specified types of diabetes, and gestational diabetes (2). Traditionally, T2DM was known as an adult-onset disease since it was usually diagnosed later in life (3). However, in recent years, the incidence of T2DM has been increasing rapidly among youth (4), prompting a growing global public health concern. T2DM in youth is further characterized by unique clinical features and demographic profiles compared to adult patients (5).

The risk factors of T2DM include many aspects, including behavioral, environmental, and social factors, and so on (6). Notably, both genetic and epigenetic factors significantly contribute to individual susceptibility. Specifically, T2DM is established as a multifactorial and polygenic disease, with numerous identified loci affecting insulin action and secretion (7). Epigenetic mechanisms (e.g., DNA methylation, histone modifications, and microRNAs) and genetic predisposition mediate the influence of environmental exposures (e.g., intrauterine malnutrition and postnatal diet) on disease risk (8). These modifications dysregulate genes critical for insulin signaling and secretion, and contribute to metabolic memory that perpetuates diabetic complications despite subsequent glycemic control (9). From an epidemiological perspective, obesity is an important factor in developing T2DM (10). Among young individuals with T2DM, obesity stands out as a particularly notable risk factor (1), with a correlation to reduced insulin sensitivity and/or secretion accompanying rising levels of adiposity (11). The clinical characteristics of T2DM among youth may range from asymptomatic hyperglycemia to diabetic ketoacidosis in up to 25% of patients or hyperglycaemic hyperosmolar syndrome (12). Furthermore, T2DM results in an increased risk of developing microvascular and macrovascular complications, substantially compromising patient quality of life (13, 14). Among youth, the prevalence of poor glycaemic control, dyslipidaemia, adiposity, and hypertension is higher in T2DM patients than in T1DM patients (15). Compared to adults with T2DM, the group of adolescents and youth is more likely to have an adverse clinical presentation, including greater insulin resistance (16, 17), increased cholesterol synthesis (18), and quicker decline of β-cell function (19). Besides, youth generally constitute a more disadvantaged group, with a higher likelihood of lacking access to opportunities for high-quality healthcare, healthy food, and physical activity (15). Considering the rise of T2DM among youth and its serious health consequences, studies suggested that T2DM developing in youth entails a greater socioeconomic burden than both T1DM in youth and T2DM in adults (20).

Clinical research for youth with T2DM is extremely important, and prevention is also of significant importance. Identifying the populations and regions most at risk of developing T2DM in youth is critical for developing targeted interventions to delay the emergence of this disease, thereby preventing the onset of complications for this demographic. This study reveals the global trends and burden of T2DM in youth, providing critical evidence to inform future research, policy development, and resource allocation against this rising health threat.

## Methods

We extracted the incidence, prevalence, and Disability-Adjusted Life Years (DALYs) of T2DM among youth aged 15–24 years from the Global Burden of Diseases, Injuries, and Risk Factors Study 2021 (GBD 2021) for analysis, spanning from 1990 to 2021. GBD 2021 provided estimates stratified by sex into female and male categories, and by age into 25 groups ranging from birth to 95 years and older, covering a total of 204 countries and territories. Based on geography, these countries and territories were divided into 21 regions. Based on the socio-demographic index (SDI), these countries and territories were subsequently categorized into 5 groups. The core responsibilities of the GBD 2021 collaborators included data collection and the statistical estimation of all burden metrics, such as incidence, prevalence, and DALYs, along with their uncertainty intervals (UIs). Our analysis was focused on the secondary analysis of these provided estimates. In this study, we centered on the age range of 15–24 years, which aligns with the WHO definition of ‘youth’ (21). Furthermore, the availability of specific data on early-onset T2DM for this youngest cohort in GBD 2021 enables a targeted investigation into this age group at the global level. According to GBD 2021, youth with T2DM aged 15–24 years were divided into two groups: 15–19 years and 20–24 years. After extracting the data from GBD 2021, our subsequent analytical work included analyzing global, regional, and national trends and burden. The global trends were analyzed by factors such as sex, age, and SDI in detail. We used point estimates with 95% UIs and assessed the trend of T2DM by estimating average annual percentage changes (AAPCs) of incidence, prevalence, and DALYs. The calculation of AAPCs was performed utilizing a linear regression model, with the independent variable being the year and the dependent variable being the log-transformed value of the estimated metric. We also conducted a separate analysis of high body mass index (BMI) as an important influencing factor. The definition of high BMI followed the protocols of GBD 2021. For individuals aged ≥18 years, we used the standard adult criteria: overweight (BMI ≥25 kg/m^2^ to <30 kg/m^2^) and obesity (BMI ≥30 kg/m^2^) (22). For adolescents aged 15–17 years, classifications were based on the sex-specific and age-specific percentiles defined by the International Obesity Task Force (IOTF) (23). Analyses were completed using R (version 4.3.1) and Joinpoint (version 5.2.0). As GBD 2021 is open source, no additional ethical approval was necessary for this analysis.

## Results

### Global trends

**Figure 1:**
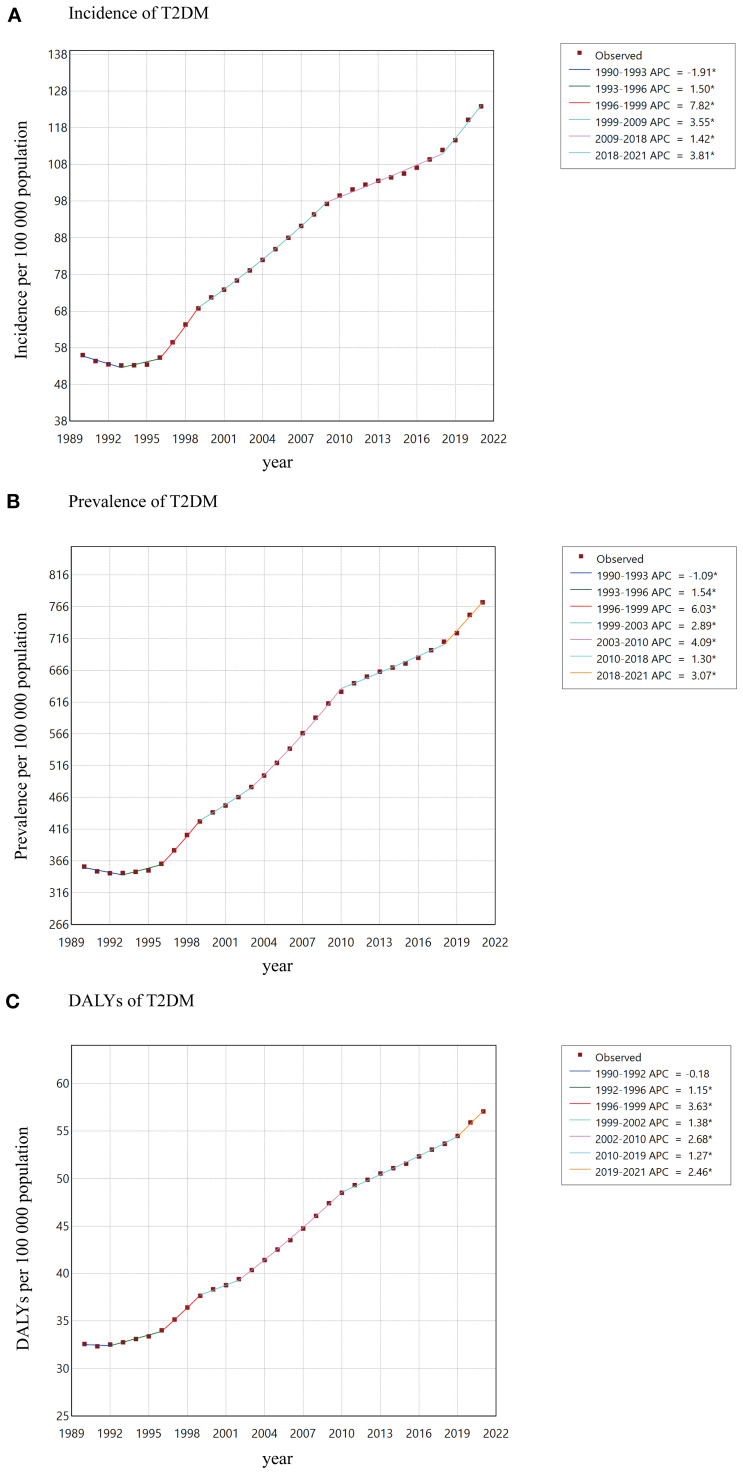
AAPCs of incidence **(A)**, prevalence **(B)**, DALYs **(C)** of T2DM among youth between 1990 and 2021.

Overall, T2DM in youth aged 15–24 years increased to varying degrees from 1990 to 2021. The global incidence increased from 56.0 per 100–000 population in 1990 to 123.9 per 100–000 population in 2021, with an AAPC of 2.62 (95% CI: 2.42 - 2.81; **Table 1**). At the global level, the incidence from 1990 to 1993 decreased with an APC of -1.91, and then from 1993 to 2021 increased with various rates (**Figure 1A**). The global prevalence increased from 357.3 per 100–000 population in 1990 to 773.0 per 100–000 population in 2021, with an AAPC of 2.54 (95% CI: 2.38 - 2.70; **Supplementary Table S1**). The global DALYs also increased from 32.6 per 100–000 population (95% UI: 25.0 - 42.6) in 1990 to 57.1 per 100–000 population (95% UI: 41.9 - 76.2) in 2021, with an AAPC of 1.84 (95% CI: 1.75 - 1.92; **Table 2**; **Figure 1C**).

**Table 1:** The incident cases, incident rates and AAPCs among youth from 1990 to 2021.

Characteristic	Incidence cases, 1990	Incidence per 100 000	Incidence cases, 2021	Incidence per 100 000	AAPCs of incidence
		Population (95% UI), 1990		Population (95% UI), 2021	(95%CI),1990-2021
Global	866750.9 (665796.1-1118873.6)	56.0 (43.0-72.3)	2338108.7 (1895924.9-2827695.3)	123.9 (100.4-149.8)	2.65 (2.42-2.81)
Sex					
Male	460032.5 (351600.9-596770.5)	58.5 (44.7-75.9)	1281868.3 (1041792.7-1545399.4)	132.5 (107.7-159.7)	2.68 (2.47-2.89)
Female	406718.4 (313519.2-521877.9)	53.4 (41.2-68.6)	1056240.4 (854368.0-1288508.4)	114.8 (92.8-140.0)	2.51 (2.35-2.66)
age					
15-19years	378664.8 (245710.1-516181.9)	72.9 (47.3-99.4)	1042699.3 (769914.7-1337642.5)	167.1 (123.4-214.4)	2.72 (2.47-2.96)
20-24years	488086.0 (344553.1-673150.3)	99.2 (70.0-136.8)	1295409.4 (972911-1664116.5)	216.9 (162.9-278.7)	2.57 (2.39-2.74)
SDI					
Low SDI	61121.3 (47346.4-78728.4)	39.3 (30.4-50.6)	340140.0 (272472.1-424686.0)	92.1 (73.8-115.0)	2.79 (2.76-2.82)
Low-middle SDI	159973.9 (123045.9-206883.7)	44.2 (34.0-57.2)	608063.0 (489086.1-763957.0)	110 (88.5-138.2)	2.98 (2.94-3.02)
Middle SDI	379219.9 (291068.7-488465.3)	69.1 (53.0-89.0)	792580.9 (643655.4-963483.3)	143.4 (116.4-174.3)	2.44 (2.24-2.65)
High-middle SDI	186947.8 (140339.8-243345.6)	65.9 (49.5-85.8)	381296.3 (311291.8-459212.8)	168.8 (137.8-203.3)	3.11 (2.85- 3.38)
High SDI	78942.6 (59191.3-102117.3)	40.3 (30.2-52.1)	214421.8 (171783.9-263112.5)	115.5 (92.6-141.8)	3.48 (3.43-3.52)
Location					
Andean Latin America	2670.9 (2008.7-3564.7)	21.7 (16.3-29.0)	8305.2 (6436.5-10763.6)	48.1 (37.3-62.3)	2.61 (2.55-2.68)
Australasia	497.8 (282.1-761.5)	10.3 (5.9-15.8)	1213.8 (747.9-1812.7)	21.2 (13.0-31.6)	2.34 (2.26-2.42)
Caribbean	6191.9 (4847.5-7926.6)	58.0 (45.4-74.2)	14789.2 (11852.7-18405.2)	130.6 (104.6-162.5)	2.66 (2.60-2.72)
Central Asia	5568.1 (4218.1-7359.8)	28.1 (21.3-37.1)	14860.0 (11709.3-18699.1)	67.2 (52.9-84.5)	2.86 (2.75-2.98)
Central Europe	1908.3 (1072.7-3011.3)	6.5 (3.7-10.3)	1570.0 (756.9-2611.1)	8.7 (4.2-14.4)	0.88 (0.74-1.01)
Central Latin America	42489.2 (33926.8-53565.8)	78.3 (62.5-98.7)	89108.2 (71000.1-111347.4)	137.0 (109.2-171.2)	1.83 (1.81-1.85)
Central Sub-Saharan Africa	7748.8 (6088.5-10002.0)	44.8 (35.2-57.8)	49251 (39050.8-61646.5)	109.6 (86.9-137.2)	2.92 (2.86-2.98)
East Asia	388497.4 (291933.2-504632.3)	104.4 (78.4-135.6)	725978.2 (591773.0-870395.8)	298.7 (243.5-358.2)	3.54 (3.25-3.83)
Eastern Europe	10080.0 (7134.7-13778.0)	21.3 (15.1-29.2)	9267.8 (6253.8-12867.8)	28.1 (19.0-39.0)	0.86 (0.66-1.06)
Eastern Sub-Saharan Africa	16463.0 (12607.4-21064.2)	26.5 (20.3-34.0)	72217.1 (57345.8-90410.7)	49.7 (39.4-62.2)	2.04 (2.01-2.07)
High-income Asia Pacific	25475.3 (19929.8-32001.9)	60.5 (47.3-76.0)	41122.1 (32992.9-51157.8)	157.5 (126.4-196)	3.13 (3.04-3.22)
High-income North America	11679.0 (6438.9-17595.8)	19.1 (10.5-28.8)	39831.9 (29890.9-51722)	55.9 (41.9-72.6)	3.50 (3.42-3.57)
North Africa and Middle East	40246.8 (30318.2-52032.8)	37.0 (27.8-47.8)	231233.4 (183700.1-282646.5)	142.5 (113.2-174.2)	4.44 (4.37-4.51)
Oceania	2755.9 (2248.0-3405.3)	131.7 (107.5-162.8)	13073.9 (10907.9-16020.6)	324.2 (270.5-397.3)	2.97 (2.89-3.06)
South Asia	175437.9 (135442.8-227932.7)	52.5 (40.5-68.1)	647713.7 (511280.7-823514.5)	123.2 (97.2-156.6)	2.77 (2.69-2.85)
Southeast Asia	42521.5 (31295.4-56704.7)	28.7 (21.1-38.2)	92378.5 (70746.2-117887.1)	54.0 (41.4-68.9)	2.15 (1.96-2.35)
Southern Latin America	2330.3 (1736.3-3002.0)	17.6 (13.1-22.7)	7014.4 (5276.9-9149.5)	45.7 (34.4-59.7)	3.13 (3.08-3.18)
Southern Sub-Saharan Africa	6761.8 (5132.7-8859.1)	39.6 (30.0-51.9)	15696.1 (12279.2-19750.3)	72.0 (56.3-90.5)	1.95 (1.90-2.00)
Tropical Latin America	15411.1 (11179.6-20969.4)	32.2 (23.4-43.8)	27509.1 (20083.3-37085)	54.4 (39.7-73.3)	1.72 (1.67-1.77)
Western Europe	40075.5 (30188.1-51875.3)	48.8 (36.7-63.1)	98113.8 (77884.6-121730.5)	136.1 (108.1-168.9)	3.31 (3.21-3.41)
Western Sub-Saharan Africa	21940.3 (16663.0-28511.5)	36.7 (27.8-47.6)	137861.3 (109115.4-171604.6)	85.4 (67.6-106.3)	2.80 (2.72-2.88)

**Table 2:** The DALYs cases, DALYs rates and AAPCs among youth from 1990 to 2021.

Characteristic	DALYs cases, 1990	DALYs per 100 000	DALYs cases, 2021	DALYs per 100 000	AAPCs of DALYs
		Population (95% UI), 1990		Population (95% UI), 2021	(95% CI), 1990-2021
Global	503832.0 (387014.4-659434.6)	32.6 (25.0-42.6)	1077230.3 (790659.3-1439357.9)	57.1 (41.9-76.2)	1.84 (1.75-1.92)
Sex					
Male	232044.2 (171817.8-309297.4)	29.5 (21.9-39.3)	540516.8 (391641.0-736210.5)	55.9 (40.5-76.1)	2.09 (2.01-2.17)
Female	271787.8 (210168.2-347965.9)	35.7 (27.6-45.7)	536713.5 (397477.4-713467.9)	58.3 (43.2-77.5)	1.61 (1.49-1.72)
Age					
15-19years	191881.7 (150362.2-252000.6)	36.9 (28.9-48.5)	377674.2 (288805.3-508653.8)	60.5(46.3-81.5)	1.61 (1.48-1.74)
20-24years	311950.3 (232200.0-419637.0)	63.4 (47.2-85.3)	699556.1 (494536.4-960375)	117.1 (82.8-160.8)	2.00 (1.89-2.11)
SDI					
Low SDI	77372.2 (61651.1-93622.4)	49.7 (39.6-60.1)	249468.4 (192169.3-320572.1)	67.5 (52.0-86.8)	0.99 (0.90-1.08)
Low-middle SDI	123405.7 (96053.8-156148.5)	34.1 (26.6-43.2)	326898.3 (242469.5-440827.3)	59.1 (43.9-79.8)	1.79 (1.66-1.92)
Middle SDI	202667.7 (153061.0-270894.7)	36.9 (27.9-49.4)	316100.2 (222132.4-434331)	57.2 (40.2-78.6)	1.44 (1.29-1.60)
High-middle SDI	71737.2 (48615.3-103457.2)	25.3 (17.1-36.5)	111681.3 (71270.4-162005.7)	49.4 (31.6-71.7)	2.22 (2.05-2.39)
High SDI	28155.1 (19474.9-40227.2)	14.4 (9.9-20.5)	72119.0 (45265.5-104900.6)	38.9 (24.4-56.5)	3.27 (3.16-3.37)
Location					
Andean Latin America	2774.2 (2279.9-3446.1)	22.5 (18.5-28)	5610.4 (4157.1-7368.8)	32.5 (24.1-42.7)	1.28 (0.68-1.88)
Australasia	165.6 (98.0-269.6)	3.4 (2.0-5.6)	336.1 (198.9-603.7)	5.9 (3.5-10.5)	1.91 (1.47-2.35)
Caribbean	6538.8 (5205.6-8102.0)	61.2 (48.7-75.9)	11236.3 (8631.0-14886.7)	99.2 (76.2-131.4)	1.61 (1.07-2.15)
Central Asia	3206.6 (2315.4-4328.5)	16.2 (11.7-21.8)	6785.8 (5046.3-8930.7)	30.7 (22.8-40.4)	2.07 (1.69-2.45)
Central Europe	1291.3 (1046.2-1734.4)	4.4 (3.6-5.9)	737.4 (623.0-963.2)	4.1 (3.4-5.3)	-0.38 (-0.85-0.09)
Central Latin America	33286.0 (26825.5-41586.8)	61.4 (49.4-76.6)	46914.0 (34503.0-64162.2)	72.1 (53.1-98.7)	0.49 (0.21-0.78)
Central Sub-Saharan Africa	10813.6 (8316.8-13671)	62.5 (48.1-79)	38063.0 (27957.5-50738.3)	84.7 (62.2-112.9)	0.91 (0.84-0.98)
East Asia	135162.8 (87763.2-201424.3)	36.3 (23.6-54.1)	196059.2 (119413.7-292504.4)	80.7 (49.1-120.4)	2.69 (2.36-3.02)
Eastern Europe	3459.0 (2110.7-5309.9)	7.3 (4.5-11.2)	2732.5 (1865.4-4012.9)	8.3 (5.7-12.2)	0.38 (-0.10-0.86)
Eastern Sub-Saharan Africa	35876.3 (28029.2-42423.1)	57.8 (45.2-68.4)	85463.8 (68225.2-109290.5)	58.8 (46.9-75.1)	0.04 (-0.09-0.16)
High-income Asia Pacific	8926.0 (6290.4-12544.3)	21.2 (14.9-29.8)	14551.0 (9084.1-21714.0)	55.7 (34.8-83.2)	3.18 (3.05-3.31)
High-income North America	4649.0 (2862.4-7599.6)	7.6 (4.7-12.4)	21326.3 (13729.7-31319.3)	29.9 (19.3-43.9)	4.57 (4.42-4.71)
North Africa and Middle East	26308.6 (20041.4-34436.8)	24.2 (18.4-31.6)	90561.4 (63711.5-125420.7)	55.8 (39.3-77.3)	2.73 (2.59-2.86)
Oceania	2001.2 (1361.2-2636.7)	95.7 (65.1-126)	6578.4 (4955.1-8717.7)	163.1 (122.9-216.2)	1.73 (1.46-2.00)
South Asia	105665.6 (76034-143012)	31.6 (22.7-42.8)	300659.7 (206817.9-424284.1)	57.2 (39.3-80.7)	1.93 (1.78-2.08)
Southeast Asia	50994.8 (40841.4-62502.4)	34.4 (27.5-42.1)	70083.5 (55483.7-88612.4)	41.0 (32.4-51.8)	0.58 (0.44-0.73)
Southern Latin America	1056.3 (936.9-1208.0)	8.0 (7.1-9.1)	2008.4 (1305.5-3035.8)	13.1 (8.5-19.8)	1.71 (1.38-2.05)
Southern Sub-Saharan Africa	7141.8 (5891.6-8811.6)	41.8 (34.5-51.6)	14190.3 (11378.5-17533.6)	65 (52.2-80.4)	1.43 (0.92-1.95)
Tropical Latin America	20669.9 (18372.2-23760.1)	43.2 (38.4-49.6)	16549.7 (12773.7-22210.3)	32.7 (25.3-43.9)	-0.81 (-1.20--0.42)
Western Europe	11381.3 (7194.2-17443.5)	13.8 (8.8-21.2)	20720.0 (12018.9-32187.1)	28.7 (16.7-44.7)	2.35 (2.27-2.43)
Western Sub-Saharan Africa	32463.4 (25607.9-39309.7)	54.2 (42.8-65.7)	126063.0 (95911.1-163594.2)	78.1 (59.4-101.4)	1.19 (1.07-1.31)

### Regional trends

The results revealed that the incident rates increased to varying degrees in all regions. The most significant increase was found in North Africa and the Middle East, with an AAPC of 4.44 (95% CI: 4.37 - 4.51). The incidence rate of increase was lowest in Eastern Europe, with an AAPC of 0.86 (95% CI: 0.66 - 1.06). Oceania had the highest incident rate (incidence 324.2 per 100–000 population, 95% UI: 270.5 - 397.3), followed by East Asia (incidence 298.7 per 100–000 population, 95% UI: 243.5 - 358.2; **Table 1**) in 2021. For DALYs, Central Europe (AAPC -0.38, 95% CI: -0.85 - 0.09) and Tropical Latin America (AAPC -0.81, 95% CI: -1.20 - -0.42) decrease from 1990 to 2021. In 2021, the DALYs were 95.7 per 100–000 population (95% CI: 65.1-126.0) in Oceania, which was the highest compared with other regions. High-income North America had the largest increase of DALYs with an AAPC of 4.57 (95% CI: 4.42 - 4.71; **Table 2**).

### National trends

**Figure 2:**
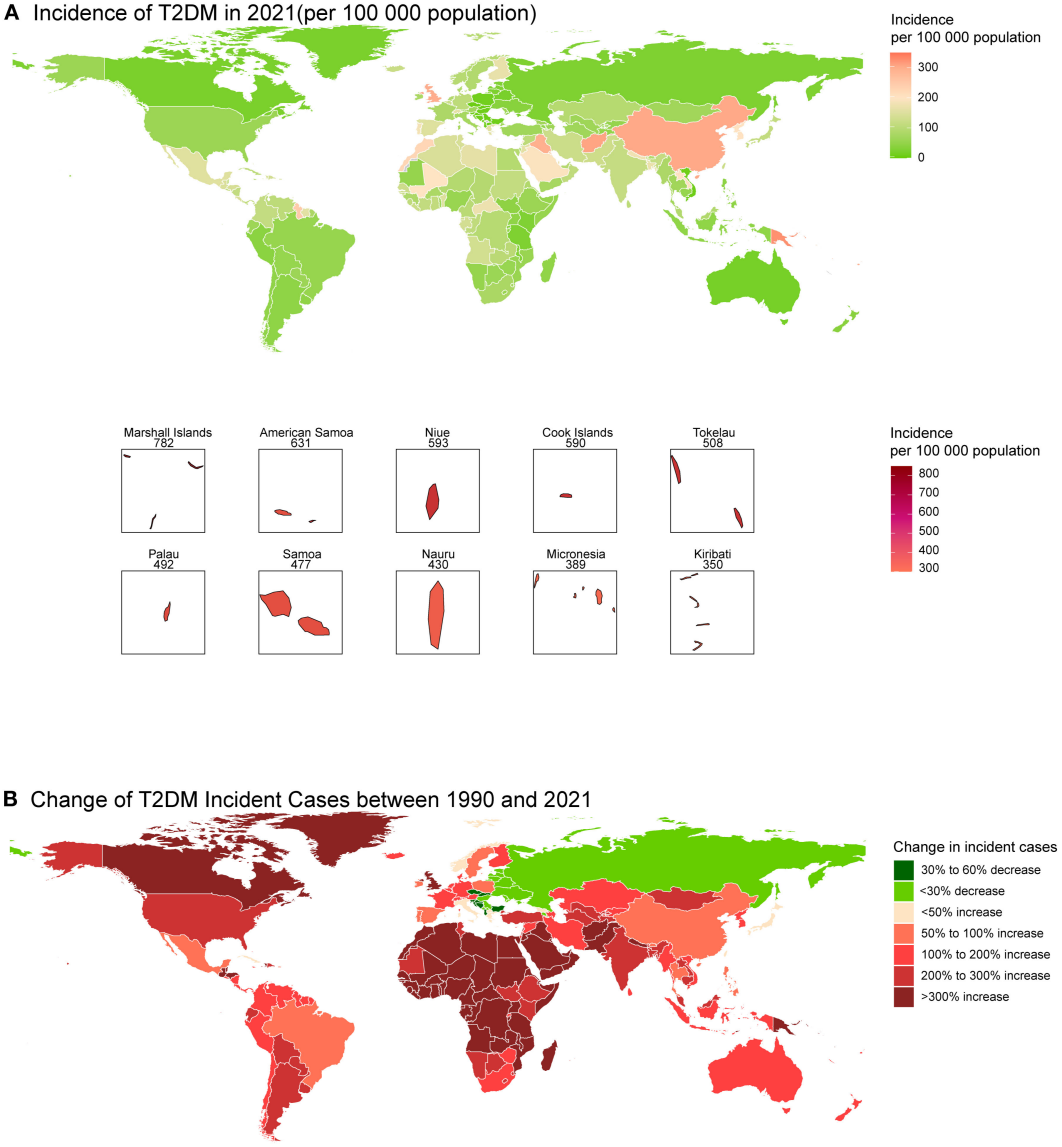
Global map of incidence of T2DM in 2021 **(A)** and the change in incident cases of T2DM among youth between 1990 and 2021 **(B)**.

The 8 countries with the highest incidence rate in 2021 were all from Oceania (e.g., Marshall Islands and American Samoa) at the national level (**Figure 2A**; **Supplementary Table S2**). With the incident cases of 710435.8 (95% CI: 578652.2 - 852861.3), China had the largest incident cases in 2021 (**Figure 2A**). The highest increase in incident cases from 1990 to 2021 occurred in Afghanistan, with a change of 1273.17% (**Supplementary Table S3**). Romania had the largest decrease, with a change in incident cases of -57.86% (**Supplementary Table S3**). Canada in North America, as well as most countries in Africa, have experienced a significant increase in T2DM cases over these 30 years, while in Eastern Europe, the number of cases has declined in most countries, with notable decreases in countries such as Latvia, Serbia, and Kosovo (**Figure 2B**).

### Global trends by gender

Upon gender comparison, the observed increase can be found both in incidence and prevalence across all genders. The incidence and prevalence of increases in males (with incidence AAPC of 2.68, 95% CI: 2.47 - 2.89; with prevalence AAPC of 2.62, 95% CI: 2.45 - 2.78) were higher than females (with incidence AAPC of 2.51, 95% CI: 2.35 - 2.66; with prevalence AAPC of 2.44, 95% CI: 2.26 - 2.62; **Table 1**). The DALYs of males (29.5, 95% CI: 21.9 - 39.3) were lower than those of females (35.7, 95% CI: 27.6 - 45.7) in 1990, but the increase was still higher over these 30 years. In 2021, the DALYs of males (DALYs 55.9, 95% UI: 40.5 - 76.1) were higher than those of females (DALYs 58.3, 95% UI: 43.2 - 77.5; **Table 2**).

### Global trends by age

The rates of incidence increased for both gender, from 72.9 (95% UI: 47.3 - 99.4) per 100–000 population to 167.1 (95% UI: 123.4 - 214.4) per 100–000 population among youth aged 15–19 years, and from 99.2 (95% UI: 70.0 - 136.8) per 100–000 population to 216.9 (95% UI: 162.9 - 278.7) per 100–000 population among youth aged 20–24 years. The incidence increased with increasing age, while the largest rate of increase could be observed in youth aged 15–19 years (AAPC 2.72, 95% CI: 2.47 - 2.96; **Table 1**, **Figure 1A**). The burden of T2DM increased more rapidly in youth aged 20–24 years (AAPC 2.00, 95% CI: 1.89 - 2.11) than in 15–19 years (AAPC 1.61, 95% CI: 1.48 - 1.74; **Figure 1B**).

### Global trends by SDI

**Figure 3:**
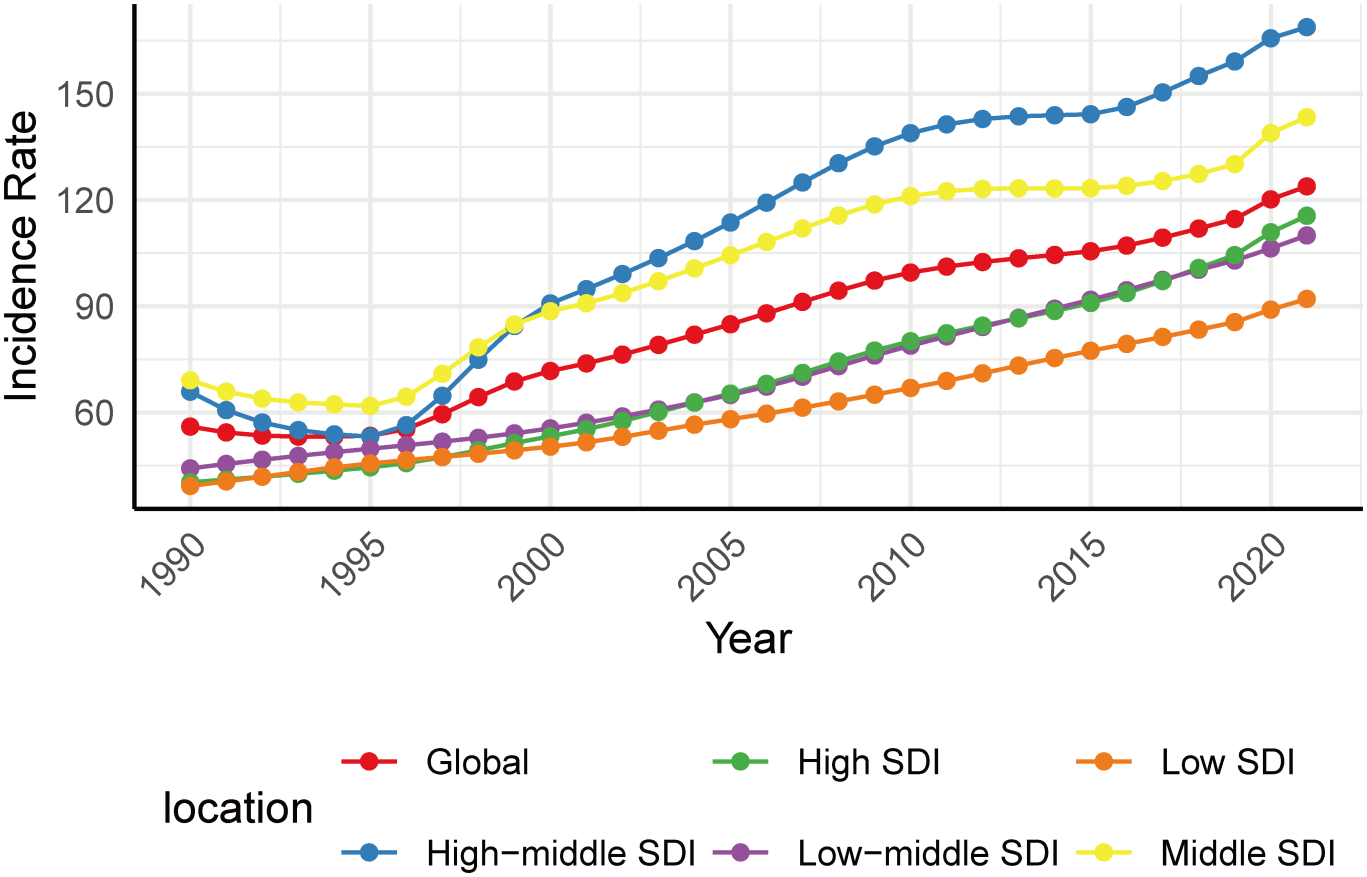
Trends in incidence of T2DM among youth by SDI between 1990 and 2021.

By SDI quintile, the incidence varied among different SDI areas. Low and high SDI areas presented lower incidence, while those middle and high-middle SDI areas presented higher incidence both in 1990 and 2021 (**Figure 3**). Despite still having a relatively low incidence rate (incidence 115.5, 95% CI: 92.6 - 141.8) in 2021, the largest increase occurred in high SDI areas (AAPC 3.48, 95% CI: 3.43 - 3.52) from 1990 to 2021. This pattern was also reflected in the increase of prevalence (AAPC 4.33, 95% CI: 4.17 - 4.49) and DALYs (AAPC 3.27, 95% CI: 3.16 - 3.37; **Table 1**) in high SDI areas.

### Risk factor

**Figure 4:**
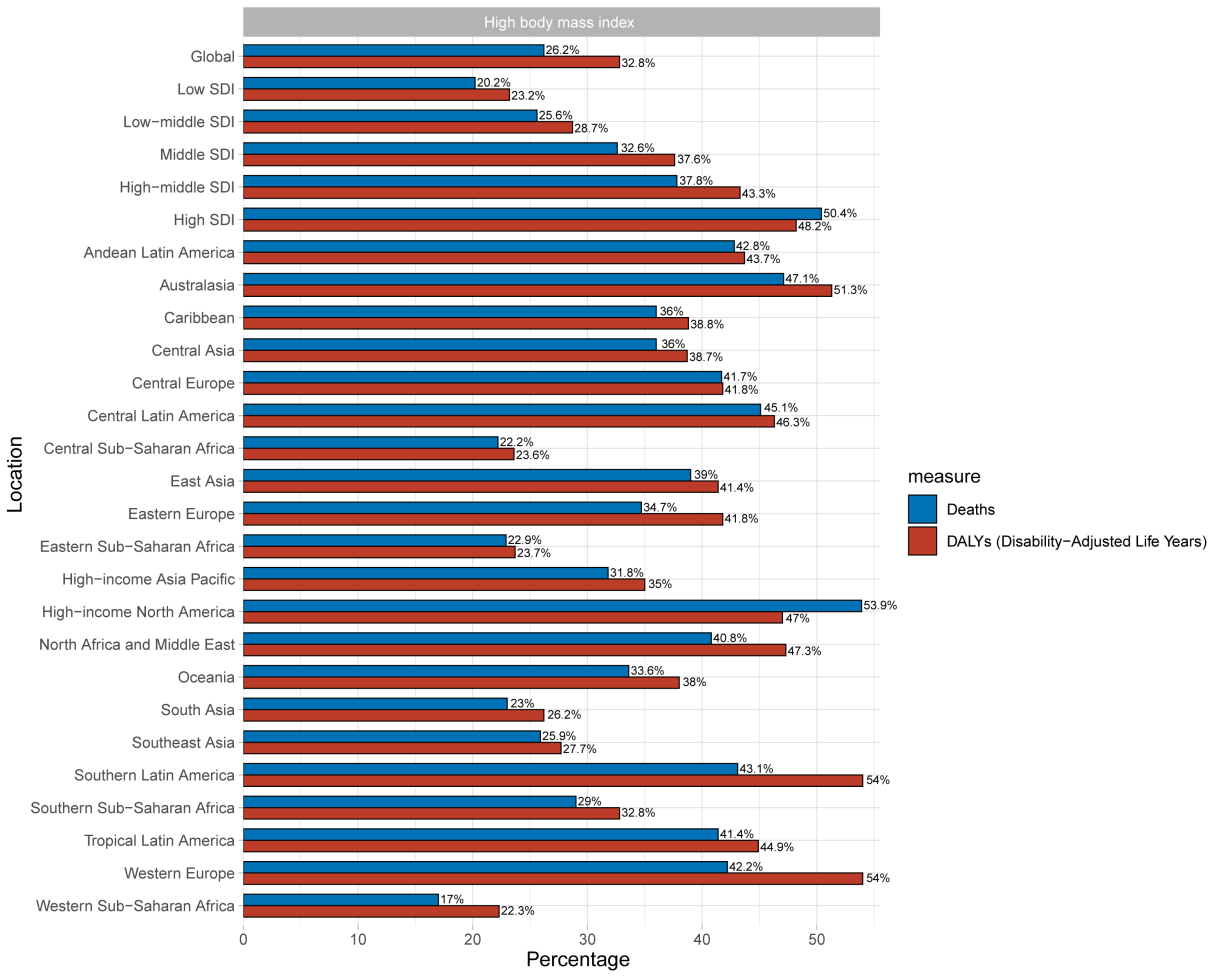
Proportion of deaths and DALYs of T2DM attributable to high BMI.

High BMI was analyzed as one of the most influential risk factors in this part. High BMI accounts for 32.8% (95% UI: 17.4% - 43.7%) of the global burden of T2DM among youth (**Figure 4**). As for SDI, the burden of T2DM increased with development in the areas, and high SDI areas bear the greatest burden, with high BMI accounting for 58.2% of T2DM burden (95% UI: 28.1% - 60.8%). South Latin America and Western Europe had the largest DALYs attributable to high BMI (**Figure 4**).

## Discussion

The study conducted a comprehensive and systematic analysis of the incidence, prevalence, and DALYs of T2DM among youth aged 15–24 years globally based on GBD 2021. In conclusion, our analysis highlights the global increase in incidence, prevalence, and DALYs among youth with T2DM. Besides, the trends and burden of T2DM youth varied by region, nation, gender, age, and development.

We found that the incidence of T2DM had doubled or more, and the number of incident cases had almost tripled among youth aged 15–24 years between 1990 and 2021. The obesity-predominant low and middle-income countries (LMICs) led to the highest incidence of T2DM in Oceania (22). Among all regions, the most substantial rise in incidence occurred in North Africa and the Middle East (AAPC of 4.44, 95% CI: 4.37 - 4.51). This explosive growth from a moderate baseline is characteristic of a rapid epidemiological transition, driven by recent, wholesale urbanization, a swift nutrition transition, and rising sedentariness (24–26). This is compounded by incomplete policy frameworks, as several countries in North Africa and the Middle East still lack operational national policies or strategies aimed at addressing risk factors like obesity and physical inactivity (27). In contrast, high-income Asia Pacific exhibited a more moderate rate of increase (AAPC of 3.13, 95% CI: 3.04 - 3.22), likely attributable to earlier implementation of public health initiatives and higher diabetes treatment coverage (28). The difference between these two socioeconomically developed regions demonstrates that the drivers of T2DM are not consistent and are heavily influenced by the stage of transition. While the former is on the steep rise of the epidemic curve, the latter may be entering a phase of stabilization at a high prevalence. At the same time, Asia has also become a global center of the T2DM epidemic, especially East Asia, which has one of the highest rates of the disease (29). This trend has been driven by economic development, shifts in dietary patterns, and rising sedentariness over the past three decades (30). Furthermore, with the great burden of T2DM affecting both youth and adults, China stands as a special case in Asia, where the economic development and dietary structure have undergone tremendous changes within just thirty years. The Global Hunger Index (GHI) of China has decreased from 13.4 in 2000 to less than 5 in 2016, and this level has been maintained since then (31). The transition in dietary patterns can also explain the high prevalence of T2DM in China. Lower incident rates were more commonly observed in Southeast European countries. Similar to this, countries with lower prevalence were mostly in Central Europe and Southeast Europe. The possibility of youth developing T2DM in Europe is relatively rare compared with other countries (32). The relatively low incidence of T2DM observed in many European countries can be partly attributed to their demographic composition, which includes a smaller proportion of youth from globally recognized high risk populations for early-onset T2DM, such as indigenous peoples of the Americas (e.g., American Indians and First Nation communities in Canada), African Americans, Hispanics, South Asians, and East Asians (15, 33). Therefore, it is necessary to take targeted measures to prevent and control T2DM among young people according to the burden in different areas.

As for age, the incidence increases with increasing age, and the incidence in youth aged 20–24 years is twice that in those aged 15–19 years. Some studies reported that few children under 14 years develop T2DM, and GBD 2021 does not have data about children aged 10–14 years with T2DM because of the assumption that all patients under 15 years were T1DM. The incidence of T2DM in prepubertal children is extremely low, with few cases under the age of 10 years, except among native Americans, indigenous Canadians, and aboriginal Australians (12). The SEARCH for Diabetes in Youth (SEARCH) study was initiated to fill the knowledge gaps and respond to emerging challenges in pediatric diabetes (4), and reported that few cases of T2DM were observed in children below 10 years from 2002 to 2012 (15). Children with T2DM are typically diagnosed around the age of 13–14 years, while girls tend to have an earlier onset (6).

Our study confirms a marked sex disparity in the global burden of T2DM (34), with young males exhibiting a disproportionately greater increase in incidence and accounting for over half of all new cases in 2021. This contrast is likely driven by a complex interplay of biological and sociobehavioral determinants. Biologically, premenopausal females are conferred protection by estrogen, which enhances insulin sensitivity and promotes a more favorable subcutaneous fat distribution, while males are more prone to visceral adiposity, a major risk factor for insulin resistance (35). From a sociocultural perspective, gender norms shape differential exposure to risk factors. Nutritional factors, such as the consumption of red meat and sugar-sweetened beverages (SSB), and physical activity patterns affect T2DM risk differently in males and females (34, 36, 37). A review suggests a trend where young males are not only exposed to more food advertising but also may be more susceptible to its influence on food preferences and purchase requests compared to females, which could lead to differential dietary patterns (38). The decline in physical activity experienced by males during early adulthood may be one of the key behavioral drivers behind the accelerated increase in their risk of T2DM. Therefore, there should be a greater focus on understanding the background of sex susceptibility and tailoring prevention strategies to effectively address the specific needs and risk profiles of each gender.

The incidence rates were lower in those areas with low SDI and high SDI, while they were higher in areas with moderate SDI. Studies indicated that the inverted U-shaped relationship was also observed in age-standardized incidence rates, in that rates increased initially with increasing SDI before eventually decreasing (39). In low SDI areas, this phenomenon may be due to the lower obesity rate. The trend of obesity rates is consistent with the incidence of T2DM, and this correlation is stressed by the fact that a majority of the youth diagnosed with T2DM were obese (40). The lower incidence in high SDI areas implies that, as the country develops, there may possibly be better access to care (29). Though with lower incidence, the largest increase was observed in high SDI areas. High SDI areas should also focus more on the fast rate to prevent it in advance. The burden is relatively more severe in moderate SDI areas compared to other areas. Research suggests that most youth with overweight or obesity living in developing countries are at high risk of developing T2DM (15). With rapid economic development, high SDI areas also experienced a great burden of non-communicable diseases, including T2DM.

Our findings on the substantial increase in T2DM burden among youth confirm the consensus from the previous GBD study (41), major external sources like the IDF Diabetes Atlas (42), the International Society for Pediatric and Adolescent Diabetes (ISPAD) registries (5), and longitudinal cohorts (e.g., the SEARCH study) (43) regarding overall trends, ethnic disparities, and the role of high BMI. However, our focused analysis on youth aged 15–24 years reveals critical insights that refine the epidemiological understanding of early-onset T2DM. We found that the most rapid rate of increase is occurring in high SDI areas, and the 15–19 years subgroup experienced the largest increase in incidence, signaling a trend toward earlier onset.

The rising burden of early-onset T2DM is particularly concerning because of its aggressive nature and the severe long-term complications it causes (5). These complications are driven not only by high blood sugar but by chronic inflammation that damages tissues throughout the body (44). Youth with T2DM face a significantly elevated risk of early-onset microvascular complications (e.g., diabetic kidney disease, retinopathy, peripheral neuropathy) and a higher lifetime risk of severe macrovascular outcomes, including cardiovascular disease and premature mortality (15). This is exemplified in the periodontal study by Fărcaş-Berechet et al. (45), which revealed significant inflammatory infiltrates (predominantly CD3-positive T-lymphocytes), microvascular alterations (CD34-positive angiogenesis), and collagen degradation in diabetic patients. Their histological and immunohistochemical findings provide mechanistic insight into how diabetes-associated inflammation contributes to tissue breakdown, a process that likely extends beyond the oral cavity to other diabetic complications. This biological plausibility stresses why the earlier onset of T2DM leads to a greater cumulative burden of disease, reflected in growing DALYs. Beyond the health metrics, this accumulating risk translates into a substantial future socioeconomic burden. Given that direct medical costs are already 2.3 times higher for people with diabetes, the longer disease duration and earlier complications of youth-onset T2DM are likely to incur even greater lifetime costs than adult-onset disease (20). Furthermore, indirect costs from potentially reduced educational attainment and productivity loss will be magnified when the disease strikes in youth. Our findings provide the critical evidence that this burden is imminent, particularly in high-SDI regions, highlighting the urgent economic imperative for aggressive preventive policies.

Consequently, the heterogeneity and gravity of this growing epidemic demand equally targeted policy responses. The rapid growth in high SDI areas calls for aggressive prevention strategies that extend beyond individual lifestyle advice to include regulatory measures like taxes on SSB (46), restrictions on marketing of unhealthy foods (47), and public investments in and expansion of structured settings that effectively increase physical activity for youth (48). The pressing need for such fiscal and regulatory interventions is highlighted by a study, which estimated that SSB taxes appear to have been effective in reducing SSB purchases and dietary intake (49). Similarly, a review focusing on children and adolescents found that the consumption of ultra-processed foods was the second most important behavioral risk factor for chronic diseases, which is due to its strong association with overweight and obesity (50). Beyond dietary measures, evidence from a meta-analysis confirms the critical role of structured settings, while school days are largely sedentary (36.7 min/hour), afterschool and physical activity programs deliver significantly higher levels of physical activity (11.7 and 20.9 min/hour of moderate to vigorous physical activity) (48). Meanwhile, in many high-middle and middle SDI areas, policy must simultaneously focus on strengthening diagnostic capacity and ensuring affordable access to essential medicines and continuous care to manage the high absolute burden and prevent debilitating complications (51). Crucially, early and aggressive intervention is required specifically to delay or prevent the debilitating complications. Furthermore, the established link between obesity and T2DM risk highlights that early screening for overweight and obese youth is a crucial universal strategy across all settings (52). From an ecological perspective, the heterogeneous effectiveness of policies like SSB taxes across countries emphasizes that their success is dependent upon implementation within a supportive environment of complementary measures.

While these policy measures are essential for immediate action, addressing the root causes and long-term outcomes of this epidemic requires further scientific inquiry. Our analysis generates pressing new questions that must guide future research. First, it is crucial to identify the specific socio-environmental drivers that are propelling the steep rise of T2DM among youth in high SDI areas. This requires research focused on these broader determinants beyond individual risks. Second, given the vulnerability of the youth aged 15–19 years, there is an urgent need for longitudinal cohort studies to track the natural history and complication rates of youth-onset T2DM.

### Strengths and limitations

The main advantage of our study is its foundation on the GBD 2021 dataset. This reliable source allows for a detailed and comprehensive assessment of global T2DM trends in young people. The study deeply analyzed the trends of T2DM among youth at the global, regional, and national levels, aiming to provide a reference point for policymakers to determine where resources are best prioritized. However, there are also some limitations to this study. First of all, the comprehensiveness of our estimates is constrained by gaps in data availability and the current scope of the GBD risk factor framework. The estimation does not cover the children aged 10–14 years, as GBD presumed all diabetes cases aged younger than 15 years were T1DM (26), an assumption based on the scarcity of population-based data for individuals younger than 15 years (39). Our risk analysis was constrained to high BMI, as it is the predominant and quantifiable risk factor for T2DM in youth captured by current models. Second, the modeling approaches, while advanced, are influenced by heterogeneity in underlying data sources. To mitigate this, GBD 2021 excludes data based solely on self-reported diabetes status without blood glucose tests to mitigate changing reporting bias over time and across locations, though this may reduce data availability (26). Variations in healthcare system infrastructure, screening practices, and vital registration completeness across countries and over time introduce inherent uncertainty (22, 53), and disparities in healthcare system infrastructure are a primary driver of this issue. A WHO survey of 160 member states found that only approximately 60% have conducted national surveys of blood glucose concentrations, and only 50% have a diabetes registry (26). This lack of standardized surveillance and testing capacity, particularly in LMICs, may contribute to significant underreporting of youth T2DM (54). Furthermore, the evidence base for screening adolescents for T2DM is limited, and its effectiveness is debated (55). The variability in recommended starting age, risk factors, and diagnostic tests (e.g., HbA1c, fasting glucose, glucose tolerance test) across leading guidelines (12, 56) may lead to inconsistent case ascertainment and misclassification between countries. To address these challenges, GBD 2021 employs advanced modelling approaches, including a Bayesian meta-regression tool (MR-BRT) and DisMod-MR to standardize disparate data sources and borrow strength across geography and time, thereby maximizing comparability (26). Finally, our attribution of T2DM burden to high BMI relies on universal cut-offs, a recognized limitation given varying ethnic-specific diabetes risk at equivalent BMIs (57, 58). For instance, South Asians face equivalent risk at a BMI of 23.9 kg/m^2^ compared to 30.0 kg/m^2^ in White populations (57). This may underestimate risk in groups like South Asians and East Asians while overestimating it in others, highlighting the need for ethnicity-specific risk estimates in future studies.

## Conclusion

While the global incidence, prevalence, and DALYs of T2DM among youth have increased over the last three decades, the trends and burden of T2DM vary by region, nation, gender, age, and development. Our findings emphasize the necessity for developing targeted public health strategies to address the heterogeneity of T2DM among youth. Global prevention and control measures for T2DM should be more personalized and differentiated, particularly in those regions and countries where the burden of this disease is growing rapidly. There is a need to enhance health education, promote a healthy lifestyle, and raise awareness of early diagnosis and treatment of T2DM among youth. Furthermore, our study highlights the influence of sex and age on T2DM, suggesting that these biological and sociocultural factors must be considered when formulating interventions. The rising cases of youth with T2DM confirm that the disease is no longer exclusive to adults, warranting further investigation in this special demographic.

## Data Availability

The original contributions presented in the study are included in the article/**Supplementary Material**. Further inquiries can be directed to the corresponding author.
