# Supplementary material for: Global, regional, and national trends and burden of diabetes mellitus type 2 among youth from 1990 to 2021: an analysis from the global burden of disease study 2021
Source: Front Endocrinol (Lausanne). 2025 Oct 22;16:1626225. doi: 10.3389/fendo.2025.1626225 (PMC12586114; doi:10.3389/fendo.2025.1626225)
Supplement: Supplementary file 1 [file Table1.docx]

**Supplementary Materials**

Supplementary Table S1. The prevalent cases, prevalent rates and AAPCs among youth from 1990 to 2021.

Supplementary Table S2. National incidence among youth in 2021.

Supplementary Table S3. Changes of national incident cases among youth between 1990 and 2021.

Supplementary Table S1. The prevalent cases, prevalent rates and AAPCs among youth from 1990 to 2021.

| Charact  eristic | Prevalent cases, 1990 | Prevalence per 100 000  population (95% UI), 1990 | Prevalent cases, 2021 | Prevalence per 100 000  population (95% UI), 2021 | AAPCs of prevalence  (95% CI),1990-2021 |
| --- | --- | --- | --- | --- | --- |
| Global | 5527767.9 (4099355.8-7248142.2) | 357.3 (265-468.5) | 14592799.6 (11652572.1-18081928.2) | 773.0 (617.3-957.8) | 2.54 (2.38-2.70) |
| Sex |  |  |  |  |  |
| Male | 2844231.8 (2112947.7-3702252.0) | 361.8 (268.8-471.0) | 7754691.4 (6174560.1-9578802.1) | 801.6 (638.2-990.1) | 2.62 (2.45-2.78) |
| Female | 2683536.1 (1978372.1-3508830.7) | 352.6 (259.9-461.0) | 6838108.2 (5451790.4-8501952.4) | 743.0 (592.4-923.8) | 2.44 (2.26-2.62) |
| Age |  |  |  |  |  |
| 15-19years | 1723777.1 (1156336.4-2345198.0) | 331.9 (222.6-451.5) | 4377049.2 (3236014.4-5651984.7) | 701.5 (518.6-905.8) | 2.45 (2.38-2.51) |
| 20-24years | 3803990.8 (2852876.3-4950386.9) | 773.0 (579.7-1006.0) | 10215750.4 (8242828.8-12542924.4) | 1710.7 (1380.3-2100.4) | 2.60 (2.45-2.76) |
| SDI |  |  |  |  |  |
| Low SDI | 463914.6 (348745.7-600570.4) | 298.0 (224.0-385.8) | 2480775.0 (1973909.6-3053855.5) | 671.5 (534.3-826.7) | 2.66 (2.63-2.68) |
| Low-middle SDI | 1154299.5 (859147.4-1491042.2) | 319.1 (237.5-412.2) | 4203345.8 (3312402.3-5271000.9) | 760.4 (599.3-953.6) | 2.83 (2.79-2.87) |
| Middle SDI | 2448054.3 (1831372.8-3160660.3) | 446.1 (333.7-575.9) | 4660095.7 (3745768.3-5725743.3) | 843.1 (677.7-1035.9) | 2.14 (1.94-2.34) |
| High-middle SDI | 1088416.0 (790610.1-1442350.2) | 383.5 (278.6-508.3) | 1945606.4 (1531096.1-2436243.7) | 861.3 (677.8-1078.6) | 2.67 (2.39-2.96) |
| High SDI | 369611.6 (235450.7-522341.4) | 188.7 (120.2-266.7) | 1293246.6 (999715.7-1616453.9) | 696.9 (538.7-871.1) | 4.33 (4.17-4.49) |
| Location |  |  |  |  |  |
| Andean Latin America | 22918.6 (17400.6-29124.3) | 186.2 (141.3-236.6) | 67732.7 (53590.8-83733.4) | 392.3 (310.4-485.0) | 2.45 (2.41-2.49) |
| Australasia | 1789.6 (672.2-3162.4) | 37.2 (14.0-65.7) | 5100.5 (2840.3-8657.0) | 88.9 (49.5-150.9) | 2.93 (2.54-3.32) |
| Caribbean | 44333.2 (32741.0-57756.8) | 415.2 (306.6-540.9) | 105942.3 (85838.7-129834.3) | 935.3 (757.8-1146.2) | 2.66 (2.63-2.70) |
| Central Asia | 38277.8 (26129.9-52225.6) | 193.0 (131.8-263.3) | 79233.6 (57745.9-103775.5) | 358.1 (261.0-469.0) | 2.01 (1.91-2.12) |
| Central Europe | 5466.6 (1449.0-11669.1) | 18.7 (5.0-40.0) | 1315.1 (6.6-5022.8) | 7.3 (0-27.7) | -3.17 (-3.38--2.96) |
| Central Latin America | 308966.2 (241390.9-387011.1) | 569.5 (444.9-713.3) | 620561.9 (492969.5-774799.4) | 954.2 (758.0-1191.4) | 1.68 (1.66-1.70) |
| Central Sub-Saharan Africa | 64757.6 (51376.6-81431.0) | 374.2 (296.8-470.5) | 372972.8 (306188.3-453443) | 829.9 (681.3-1008.9) | 2.61 (2.52-2.69) |
| East Asia | 2324510.4 (1705676.4-3025443.9) | 624.6 (458.3-812.9) | 3709958.4 (3000143.5-4493325.8) | 1526.7 (1234.6-1849.0) | 3.02 (2.68-3.35) |
| Eastern Europe | 50545.6 (29355.2-74916.1) | 107.0 (62.2-158.6) | 24786.1 (10801.7-43222.8) | 75.1 (32.7-131.0) | -1.16 (-1.30--1.02) |
| Eastern Sub-Saharan Africa | 108461.1 (73770.5-149840.3) | 174.8 (118.9-241.5) | 509637.5 (383693.2-652984.2) | 350.4 (263.8-449.0) | 2.26 (2.17-2.35) |
| High-income Asia Pacific | 111315.0 (72872.1-154331.0) | 264.2 (173.0-366.3) | 270558.8 (210384.5-338342.6) | 1036.4 (805.9-1296.1) | 4.50 (4.36-4.63) |
| High-income North America | 41986.5 (8277.5-90866.2) | 68.6 (13.5-148.5) | 383614.8 (295343-480645.1) | 538.2 (414.3-674.3) | 6.92 (6.75-7.09) |
| North Africa and Middle East | 262860.3 (187328.6-348976.3) | 241.4 (172.0-320.4) | 1300919.2 (1004386.2-1608274.4) | 801.6 (618.9-990.9) | 3.95 (3.88-4.01) |
| Oceania | 17056.7 (13821.1-20893.1) | 815.4 (660.7-998.8) | 73316.6 (61216.3-88160.4) | 1818.2 (1518.1-2186.3) | 2.65 (2.57-2.72) |
| South Asia | 1302553.0 (974598.9-1675912.9) | 389.4 (291.4-501.1) | 4605364.3 (3587280.1-5788299.5) | 875.8 (682.2-1100.7) | 2.63 (2.59-2.67) |
| Southeast Asia | 289367.7 (196197.2-391224.9) | 195.0 (132.2-263.7) | 582238.9 (433434.2-746891.5) | 340.5 (253.5-436.8) | 1.83 (1.29-2.38) |
| Southern Latin America | 1649.3 (479.2-3832.0) | 12.5 (3.6-28.9) | 24834.7 (13923.7-38971.8) | 161.9 (90.8-254.1) | 8.63 (8.45-8.81) |
| Southern Sub-Saharan Africa | 51548.4 (37303.9-67300.1) | 301.7 (218.4-393.9) | 120648.9 (93954.8-149287.5) | 553.1 (430.7-684.3) | 1.96 (1.91-2.01) |
| Tropical Latin America | 93479.7 (60136.2-131091.5) | 195.3 (125.7-273.9) | 158070.3 (105825.2-216956.4) | 312.5 (209.2-429.0) | 1.53 (1.47-1.58) |
| Western Europe | 179416.7 (118070.1-248032.8) | 218.3 (143.6-301.7) | 390331.7 (270486.3-524500.9) | 541.6 (375.3-727.7) | 2.96 (2.83-3.08) |
| Western Sub-Saharan Africa | 206507.8 (159901.7-259290.5) | 345.1 (267.2-433.3) | 1185660.4 (945244.9-1449521.2) | 734.8 (585.8-898.3) | 2.48 (2.42-2.53) |

Supplementary Table S2. National incidence among youth in 2021.

| Location | Incidence per 100 000 population, 2021 | 95% UI (upper) | 95% UI (lower) |
| --- | --- | --- | --- |
| Afghanistan | 307.2 | 375.5 | 248.1 |
| Albania | 8.6 | 14.4 | 4.0 |
| Algeria | 143.4 | 176.6 | 112.3 |
| American Samoa | 630.9 | 782.8 | 500.1 |
| Andorra | 117.7 | 148.1 | 89.2 |
| Angola | 133.5 | 167.6 | 100.9 |
| Antigua and Barbuda | 124.0 | 159.8 | 96.5 |
| Argentina | 44.7 | 58.9 | 33.9 |
| Armenia | 48.6 | 66.2 | 35.9 |
| Australia | 17.7 | 27.4 | 9.4 |
| Austria | 67.8 | 85.8 | 52.1 |
| Azerbaijan | 64.3 | 81.8 | 48.8 |
| Bahamas | 132.1 | 172.2 | 103.5 |
| Bahrain | 176.6 | 227.3 | 133.4 |
| Bangladesh | 168.4 | 207.9 | 132.3 |
| Barbados | 120.0 | 153.8 | 93.5 |
| Belarus | 19.8 | 28.2 | 13.2 |
| Belgium | 119.7 | 150.3 | 93.1 |
| Belize | 111.4 | 140.9 | 89.0 |
| Benin | 151.5 | 191.3 | 119.2 |
| Bermuda | 83.7 | 108.2 | 65.8 |
| Bhutan | 93.1 | 118.9 | 74.6 |
| Bolivia (Plurinational State of Bolivia) | 49.4 | 65.2 | 37.9 |
| Bosnia and Herzegovina | 8.2 | 14.9 | 2.9 |
| Botswana | 60.9 | 78.4 | 47.1 |
| Brazil | 54.1 | 73.1 | 39.2 |
| Brunei Darussalam | 232.2 | 295.6 | 180.3 |
| Bulgaria | 8.5 | 15.2 | 3.0 |
| Burkina Faso | 81.0 | 100.9 | 63.4 |
| Burundi | 47.5 | 59.8 | 37.4 |
| Cabo Verde | 157.7 | 198.2 | 121.0 |
| Cambodia | 53.2 | 68.4 | 41.1 |
| Cameroon | 100.5 | 126.1 | 79.1 |
| Canada | 21.0 | 37.1 | 8.6 |
| Central African Republic | 173.3 | 221.8 | 137.7 |
| Chad | 92.7 | 115.5 | 73.8 |
| Chile | 48.6 | 64.2 | 36.1 |
| China | 303.6 | 364.4 | 247.2 |
| Colombia | 114.2 | 145.6 | 90.8 |
| Comoros | 80.2 | 103.8 | 61.8 |
| Congo | 107.4 | 135.3 | 84.9 |
| Cook Islands | 590.1 | 726.2 | 469.3 |
| Costa Rica | 143.9 | 186.7 | 111.8 |
| Côte d'Ivoire | 110.4 | 136.5 | 86.9 |
| Croatia | 6.6 | 11.2 | 2.6 |
| Cuba | 104.4 | 135.0 | 81.7 |
| Cyprus | 112.9 | 143.5 | 84.3 |
| Czechia | 7.3 | 12.4 | 3.2 |
| Democratic People's Republic of Korea | 204.5 | 250.0 | 164.4 |
| Democratic Republic of the Congo | 96.5 | 122.6 | 75.5 |
| Denmark | 85.4 | 109.4 | 64.3 |
| Djibouti | 50.1 | 64.7 | 39.5 |
| Dominica | 151.5 | 195.7 | 120.7 |
| Dominican Republic | 135.4 | 170.5 | 107.7 |
| Ecuador | 63.4 | 80.3 | 48.9 |
| Egypt | 93.3 | 117.4 | 71.9 |
| El Salvador | 107.8 | 136.0 | 84.2 |
| Equatorial Guinea | 130.8 | 166.0 | 104.1 |
| Eritrea | 87.8 | 112.1 | 67.4 |
| Estonia | 27.1 | 39.5 | 17.2 |
| Eswatini | 71.5 | 90.8 | 55.6 |
| Ethiopia | 60.3 | 76.7 | 46.6 |
| Fiji | 292.2 | 360.2 | 238.1 |
| Finland | 165.6 | 214.3 | 123.5 |
| France | 82.3 | 106.7 | 62.6 |
| Gabon | 136.3 | 177.4 | 108.5 |
| Gambia | 101.2 | 127.1 | 79.5 |
| Georgia | 68.0 | 88.6 | 50.6 |
| Germany | 104.7 | 130.0 | 80.1 |
| Ghana | 110.0 | 137.2 | 84.6 |
| Greece | 156.2 | 198.5 | 123.3 |
| Greenland | 21.5 | 31.1 | 12.7 |
| Grenada | 145.1 | 189.2 | 111.9 |
| Guam | 276.4 | 340.3 | 220.4 |
| Guatemala | 144.7 | 185.1 | 114.6 |
| Guinea | 83.3 | 105.4 | 65.2 |
| Guinea-Bissau | 121.1 | 154.0 | 96.0 |
| Guyana | 246.1 | 309.8 | 191.6 |
| Haiti | 140.3 | 179.2 | 109.5 |
| Honduras | 139.4 | 176.9 | 107.3 |
| Hungary | 9.2 | 15.2 | 4.7 |
| Iceland | 120.0 | 151.5 | 93.8 |
| India | 116.1 | 148.5 | 90.3 |
| Indonesia | 46.2 | 61.3 | 34.2 |
| Iran (Islamic Republic of Iran) | 75.5 | 99.1 | 57.4 |
| Iraq | 293.9 | 370.3 | 229.8 |
| Ireland | 81.5 | 106.4 | 61.0 |
| Israel | 69.8 | 89.0 | 51.1 |
| Italy | 72.7 | 105.8 | 46.1 |
| Jamaica | 85.4 | 111.3 | 66.7 |
| Japan | 111.8 | 142.6 | 86.4 |
| Jordan | 160.6 | 202.2 | 124.3 |
| Kazakhstan | 93.8 | 119.1 | 72.4 |
| Kenya | 26.7 | 34.7 | 20.1 |
| Kiribati | 350.3 | 426.7 | 288.0 |
| Kuwait | 234.9 | 293.4 | 184.2 |
| Kyrgyzstan | 51.4 | 67.9 | 38.6 |
| Lao People's Democratic Republic | 67.7 | 86.6 | 52.6 |
| Latvia | 27.8 | 39.7 | 18.3 |
| Lebanon | 184.8 | 230.1 | 141.0 |
| Lesotho | 55.4 | 71.9 | 42.2 |
| Liberia | 125.6 | 161.0 | 98.7 |
| Libya | 161.2 | 203.0 | 122.8 |
| Lithuania | 25.6 | 37.7 | 16.8 |
| Luxembourg | 120.7 | 154.2 | 93.9 |
| Madagascar | 46.5 | 61.2 | 36.3 |
| Malawi | 32.8 | 41.4 | 25.7 |
| Malaysia | 89.1 | 115.7 | 68.6 |
| Maldives | 62.1 | 79.2 | 47.6 |
| Mali | 199.3 | 253.0 | 153.7 |
| Malta | 148.8 | 191.1 | 113.9 |
| Marshall Islands | 782.3 | 966.2 | 635.9 |
| Mauritania | 51.8 | 64.4 | 40.7 |
| Mauritius | 121.3 | 158.5 | 92.4 |
| Mexico | 153.1 | 194.1 | 119.8 |
| Micronesia (Federated States of Micronesia) | 388.9 | 475.3 | 316.5 |
| Monaco | 118.5 | 150.4 | 91.1 |
| Mongolia | 68.3 | 87.0 | 52.6 |
| Montenegro | 9.4 | 15.4 | 4.6 |
| Morocco | 225.0 | 288.2 | 171.3 |
| Mozambique | 57.2 | 73.8 | 44.2 |
| Myanmar | 82.0 | 107.3 | 62.0 |
| Namibia | 52.8 | 67.7 | 40.1 |
| Nauru | 429.6 | 524.5 | 360.0 |
| Nepal | 177.0 | 220.6 | 140.7 |
| Netherlands | 92.3 | 117.8 | 68.8 |
| New Zealand | 37.4 | 49.4 | 27.6 |
| Nicaragua | 114.2 | 147.2 | 91.0 |
| Niger | 91.9 | 115.6 | 72.8 |
| Nigeria | 57.3 | 73.6 | 44.5 |
| Niue | 592.7 | 742.5 | 472.0 |
| North Macedonia | 7.4 | 13.6 | 2.1 |
| Northern Mariana Islands | 273.5 | 337.0 | 212.5 |
| Norway | 96.9 | 129.8 | 70.2 |
| Oman | 107.8 | 139.3 | 79.2 |
| Pakistan | 126.4 | 161.4 | 99.1 |
| Palau | 492.4 | 595.1 | 400.0 |
| Palestine | 99.0 | 126.2 | 77.3 |
| Panama | 109.9 | 141.1 | 86.2 |
| Papua New Guinea | 325.3 | 399.4 | 270.7 |
| Paraguay | 62.1 | 81.2 | 48.3 |
| Peru | 39.5 | 52.0 | 30.5 |
| Philippines | 42.6 | 55.4 | 32.6 |
| Poland | 11.6 | 18.9 | 6.0 |
| Portugal | 183.8 | 229.8 | 140.7 |
| Puerto Rico | 134.8 | 177.6 | 101.1 |
| Qatar | 169.6 | 218.9 | 125.8 |
| Republic of Korea | 259.6 | 320.8 | 209.8 |
| Republic of Moldova | 36.1 | 50.2 | 24.0 |
| Romania | 5.9 | 10.1 | 2.7 |
| Russian Federation | 26.3 | 37.1 | 17.5 |
| Rwanda | 35.4 | 45.4 | 27.9 |
| Saint Kitts and Nevis | 126.1 | 162.7 | 97.9 |
| Saint Lucia | 164.3 | 206.9 | 125.8 |
| Saint Vincent and the Grenadines | 159.2 | 208.8 | 123.8 |
| Samoa | 476.7 | 591.1 | 381.5 |
| San Marino | 118.8 | 152.2 | 91.0 |
| Sao Tome and Principe | 137.6 | 171.9 | 107.0 |
| Saudi Arabia | 192.7 | 244.4 | 147.9 |
| Senegal | 127.8 | 161.0 | 102.1 |
| Serbia | 7.3 | 12.7 | 3.1 |
| Seychelles | 124.8 | 165.5 | 94.1 |
| Sierra Leone | 108.0 | 139.2 | 86.0 |
| Singapore | 170.6 | 216.1 | 133.0 |
| Slovakia | 5.8 | 10.4 | 2.2 |
| Slovenia | 6.6 | 11.7 | 3.0 |
| Solomon Islands | 236.3 | 289.4 | 196.9 |
| Somalia | 54.9 | 70.6 | 42.5 |
| South Africa | 79.9 | 101.1 | 62.2 |
| South Sudan | 44.3 | 57.2 | 33.8 |
| Spain | 150.9 | 189.1 | 117.6 |
| Sri Lanka | 103.8 | 132.5 | 79.2 |
| Sudan | 113.2 | 141.6 | 88.2 |
| Suriname | 159.4 | 205.9 | 122.2 |
| Sweden | 89.6 | 120.6 | 64.6 |
| Switzerland | 163.3 | 207.1 | 126.0 |
| Syrian Arab Republic | 126.2 | 159.3 | 96.7 |
| Taiwan (Province of China) | 124.8 | 154.8 | 95.9 |
| Tajikistan | 49.2 | 64.7 | 37.3 |
| Thailand | 67.7 | 87.3 | 53.0 |
| Timor-Leste | 62.1 | 78.8 | 47.6 |
| Togo | 64.1 | 79.2 | 51.0 |
| Tokelau | 508.3 | 622.8 | 411.9 |
| Tonga | 324.1 | 405.7 | 264.6 |
| Trinidad and Tobago | 140.6 | 181.6 | 106.9 |
| Tunisia | 140.5 | 179.4 | 106.7 |
| Türkiye | 87.9 | 109.5 | 68.7 |
| Turkmenistan | 65.8 | 83.6 | 51.3 |
| Tuvalu | 291.2 | 358.9 | 233.2 |
| Uganda | 44.9 | 57.1 | 34.8 |
| Ukraine | 35.8 | 48.5 | 24.8 |
| United Arab Emirates | 94.8 | 121.0 | 70.9 |
| United Kingdom | 299.4 | 365.6 | 239.3 |
| United Republic of Tanzania | 37.4 | 47.4 | 29.3 |
| United States of America | 59.4 | 76.6 | 44.8 |
| United States Virgin Islands | 143.1 | 182.9 | 107.8 |
| Uruguay | 45.1 | 60.4 | 32.9 |
| Uzbekistan | 65.3 | 83.9 | 50.1 |
| Vanuatu | 310.8 | 377.3 | 251.6 |
| Venezuela (Bolivarian Republic of Venezuela) | 104.7 | 132.1 | 82.2 |
| Viet Nam | 41.2 | 53.3 | 31.3 |
| Yemen | 75.5 | 96.0 | 57.9 |
| Zambia | 101.0 | 127.2 | 79.0 |
| Zimbabwe | 55.7 | 70.4 | 43.7 |

Supplementary Table S3. Changes of national incident cases among youth between 1990 and 2021.

| Region | Incident cases, 1900 | Incident cases, 2021 | Changes of cases | Percent of changes |
| --- | --- | --- | --- | --- |
| Afghanistan | 2382.8 | 32719.9 | 1273.2% | >300% increase |
| Albania | 71.4 | 45.4 | -36.4% | 30% to 60% decrease |
| Algeria | 3595.7 | 14581.4 | 305.5% | >300% increase |
| American Samoa | 32.3 | 87.9 | 172.1% | 100% to 200% increase |
| Andorra | 6.3 | 15.3 | 144.3% | 100% to 200% increase |
| Angola | 1774.3 | 14094.7 | 694.4% | >300% increase |
| Antigua and Barbuda | 10.8 | 24.4 | 125.3% | 100% to 200% increase |
| Argentina | 1451.8 | 4782.9 | 229.4% | 200% to 300% increase |
| Armenia | 268.6 | 262.1 | -2.4% | <30% decrease |
| Australia | 352.6 | 835.5 | 137.0% | 100% to 200% increase |
| Austria | 444.3 | 957.8 | 115.5% | 100% to 200% increase |
| Azerbaijan | 557.3 | 1417.6 | 154.4% | 100% to 200% increase |
| Bahamas | 48.5 | 131.0 | 170.2% | 100% to 200% increase |
| Bahrain | 62.6 | 593.0 | 846.8% | >300% increase |
| Bangladesh | 20501.5 | 77128.6 | 276.2% | 200% to 300% increase |
| Barbados | 41.8 | 68.5 | 63.9% | 50% to 100% increase |
| Barbuda | 10.8 | 24.4 | 125.3% | 100% to 200% increase |
| Belarus | 350.5 | 281.0 | -19.8% | <30% decrease |
| Belgium | 1088.9 | 2350.9 | 115.9% | 100% to 200% increase |
| Belize | 28.0 | 147.0 | 425.5% | >300% increase |
| Benin | 635.8 | 6632.8 | 943.2% | >300% increase |
| Bermuda | 5.1 | 7.7 | 49.5% | <50% increase |
| Bhutan | 104.8 | 190.6 | 81.9% | 50% to 100% increase |
| Bolivia (Plurinational State of Bolivia) | 440.6 | 1598.9 | 262.9% | 200% to 300% increase |
| Bosnia and Herzegovina | 72.4 | 44.2 | -39.0% | 30% to 60% decrease |
| Botswana | 120.9 | 398.4 | 229.5% | 200% to 300% increase |
| Brazil | 15042.8 | 26272.7 | 74.7% | 50% to 100% increase |
| Brunei Darussalam | 63.4 | 246.3 | 288.1% | 200% to 300% increase |
| Bulgaria | 153.7 | 82.6 | -46.3% | 30% to 60% decrease |
| Burkina Faso | 773.9 | 5887.4 | 660.7% | >300% increase |
| Burundi | 448.9 | 2050.4 | 356.7% | >300% increase |
| Cabo Verde | 58.6 | 236.7 | 303.9% | >300% increase |
| Cambodia | 666.0 | 2462.3 | 269.7% | 200% to 300% increase |
| Cameroon | 1141.2 | 10434.3 | 814.3% | >300% increase |
| Canada | 253.1 | 1360.1 | 437.3% | >300% increase |
| Central African Republic | 565.3 | 3143.9 | 456.1% | >300% increase |
| Chad | 675.9 | 5409.3 | 700.3% | >300% increase |
| Chile | 775.5 | 1906.5 | 145.8% | 100% to 200% increase |
| China | 380012.5 | 710435.8 | 87.0% | 50% to 100% increase |
| Colombia | 6292.9 | 13671.2 | 117.2% | 100% to 200% increase |
| Comoros | 52.8 | 176.8 | 234.6% | 200% to 300% increase |
| Congo | 371.4 | 1791.0 | 382.2% | >300% increase |
| Cook Islands | 13.0 | 24.1 | 85.4% | 50% to 100% increase |
| Costa Rica | 533.7 | 1573.7 | 194.9% | 100% to 200% increase |
| Croatia | 68.5 | 43.5 | -36.5% | 30% to 60% decrease |
| Cuba | 1858.3 | 2038.2 | 9.7% | <50% increase |
| Cyprus | 100.0 | 244.4 | 144.4% | 100% to 200% increase |
| Czechia | 172.4 | 114.7 | -33.5% | 30% to 60% decrease |
| Democratic People's Republic of Korea | 4742.5 | 11154.3 | 135.2% | 100% to 200% increase |
| Democratic Republic of the Congo | 4807.8 | 28736.4 | 497.7% | >300% increase |
| Denmark | 423.1 | 896.3 | 111.8% | 100% to 200% increase |
| Djibouti | 33.4 | 178.4 | 433.9% | >300% increase |
| Dominica | 17.0 | 24.9 | 46.9% | <50% increase |
| Dominican Republic | 1221.6 | 3868.0 | 216.6% | 200% to 300% increase |
| Ecuador | 872.3 | 3080.5 | 253.2% | 200% to 300% increase |
| Egypt | 3409.6 | 27756.2 | 714.1% | >300% increase |
| El Salvador | 841.2 | 1885.2 | 124.1% | 100% to 200% increase |
| Equatorial Guinea | 63.2 | 716.2 | 1032.5% | >300% increase |
| Eritrea | 382.9 | 1831.1 | 378.2% | >300% increase |
| Estonia | 56.5 | 54.8 | -3.0% | <30% decrease |
| Eswatini | 85.2 | 263.0 | 208.8% | 200% to 300% increase |
| Ethiopia | 6453.1 | 22729.3 | 252.2% | 200% to 300% increase |
| Fiji | 321.2 | 699.4 | 117.8% | 100% to 200% increase |
| Finland | 697.4 | 1518.7 | 117.8% | 100% to 200% increase |
| France | 4179.4 | 10057.8 | 140.7% | 100% to 200% increase |
| Gabon | 166.7 | 768.8 | 361.1% | >300% increase |
| Gambia | 116.3 | 824.3 | 608.9% | >300% increase |
| Georgia | 364.2 | 423.4 | 16.2% | <50% increase |
| Germany | 5772.2 | 13221.4 | 129.1% | 100% to 200% increase |
| Ghana | 1970.2 | 11555.6 | 486.5% | >300% increase |
| Greece | 1633.5 | 2411.4 | 47.6% | <50% increase |
| Greenland | 0.3 | 2.4 | 605.0% | >300% increase |
| Grenada | 18.8 | 36.9 | 96.4% | 50% to 100% increase |
| Guam | 50.5 | 99.4 | 96.8% | 50% to 100% increase |
| Guatemala | 1267.6 | 7157.4 | 464.6% | >300% increase |
| Guinea | 539.9 | 3607.2 | 568.2% | >300% increase |
| Guinea-Bissau | 161.4 | 814.1 | 404.4% | >300% increase |
| Guyana | 253.4 | 514.1 | 102.9% | 100% to 200% increase |
| Haiti | 1309.0 | 5248.0 | 300.9% | >300% increase |
| Honduras | 896.1 | 4438.5 | 395.3% | >300% increase |
| Hungary | 191.1 | 134.1 | -29.8% | <30% decrease |
| Iceland | 31.1 | 81.8 | 163.4% | 100% to 200% increase |
| India | 133883.2 | 461939.4 | 245.0% | 200% to 300% increase |
| Indonesia | 15498.8 | 31965.7 | 106.2% | 100% to 200% increase |
| Iran (Islamic Republic of Iran) | 4538.5 | 13368.0 | 194.5% | 100% to 200% increase |
| Iraq | 5869.1 | 36784.9 | 526.8% | >300% increase |
| Ireland | 437.7 | 801.3 | 83.1% | 50% to 100% increase |
| Israel | 721.5 | 1572.7 | 118.0% | 100% to 200% increase |
| Italy | 5838.6 | 6320.9 | 8.3% | <50% increase |
| Jamaica | 266.6 | 606.0 | 127.3% | 100% to 200% increase |
| Japan | 15681.5 | 19633.5 | 25.2% | <50% increase |
| Jordan | 714.4 | 6081.2 | 751.2% | >300% increase |
| Kazakhstan | 1624.1 | 3849.3 | 137.0% | 100% to 200% increase |
| Kenya | 1399.8 | 4609.8 | 229.3% | 200% to 300% increase |
| Kiribati | 37.7 | 123.8 | 228.8% | 200% to 300% increase |
| Kuwait | 338.7 | 1957.9 | 478.0% | >300% increase |
| Kyrgyzstan | 330.8 | 911.7 | 175.6% | 100% to 200% increase |
| Lao People's Democratic Republic | 366.2 | 1413.1 | 285.9% | 200% to 300% increase |
| Latvia | 106.8 | 75.3 | -29.5% | <30% decrease |
| Lebanon | 451.8 | 2190.3 | 384.8% | >300% increase |
| Lesotho | 91.2 | 338.8 | 271.4% | 200% to 300% increase |
| Liberia | 311.4 | 2259.1 | 625.4% | >300% increase |
| Libya | 578.3 | 2810.7 | 386.0% | >300% increase |
| Lithuania | 105.9 | 105.7 | -0.1% | <30% decrease |
| Luxembourg | 39.7 | 129.1 | 225.3% | 200% to 300% increase |
| Madagascar | 942.8 | 4464.5 | 373.5% | >300% increase |
| Malawi | 574.0 | 2333.9 | 306.6% | >300% increase |
| Malaysia | 2098.7 | 7208.7 | 243.5% | 200% to 300% increase |
| Maldives | 18.3 | 62.0 | 238.9% | 200% to 300% increase |
| Mali | 1870.0 | 15927.6 | 751.8% | >300% increase |
| Malta | 31.8 | 94.7 | 197.3% | 100% to 200% increase |
| Marshall Islands | 36.9 | 129.7 | 251.8% | 200% to 300% increase |
| Mauritania | 188.3 | 744.1 | 295.1% | 200% to 300% increase |
| Mauritius | 123.5 | 313.4 | 153.8% | 100% to 200% increase |
| Mexico | 28446.3 | 50817.5 | 78.6% | 50% to 100% increase |
| Micronesia (Federated States of Micronesia) | 46.4 | 121.9 | 162.8% | 100% to 200% increase |
| Monaco | 2.3 | 6.2 | 171.8% | 100% to 200% increase |
| Mongolia | 168.9 | 518.4 | 207.0% | 200% to 300% increase |
| Montenegro | 13.5 | 11.0 | -18.5% | <30% decrease |
| Morocco | 4186.3 | 21025.0 | 402.2% | >300% increase |
| Mozambique | 800.7 | 6052.8 | 656.0% | >300% increase |
| Myanmar | 5127.9 | 12501.1 | 143.8% | 100% to 200% increase |
| Namibia | 129.4 | 393.9 | 204.5% | 200% to 300% increase |
| Nauru | 5.3 | 14.7 | 180.1% | 100% to 200% increase |
| Nepal | 3510.5 | 16732.4 | 376.6% | >300% increase |
| Netherlands | 1271.4 | 2777.5 | 118.5% | 100% to 200% increase |
| New Zealand | 145.2 | 378.3 | 160.5% | 100% to 200% increase |
| Nicaragua | 722.9 | 2149.1 | 197.3% | 100% to 200% increase |
| Niger | 929.9 | 7744.9 | 732.8% | >300% increase |
| Nigeria | 8717.7 | 44679.1 | 412.5% | >300% increase |
| Niue | 1.2 | 2.4 | 103.9% | 100% to 200% increase |
| North Macedonia | 27.0 | 27.4 | 1.5% | <50% increase |
| Northern Mariana Islands | 17.3 | 31.3 | 81.3% | 50% to 100% increase |
| Norway | 648.4 | 956.4 | 47.5% | <50% increase |
| Oman | 169.8 | 999.8 | 488.8% | >300% increase |
| Pakistan | 17438.1 | 91722.7 | 426.0% | >300% increase |
| Palau | 9.0 | 17.3 | 91.2% | 50% to 100% increase |
| Palestine | 233.7 | 1636.5 | 600.2% | >300% increase |
| Panama | 413.2 | 1211.5 | 193.2% | 100% to 200% increase |
| Papua New Guinea | 1712.9 | 9934.4 | 480.0% | >300% increase |
| Paraguay | 368.3 | 1236.3 | 235.7% | 200% to 300% increase |
| Peru | 1358.0 | 3625.7 | 167.0% | 100% to 200% increase |
| Philippines | 7116.5 | 13857.6 | 94.7% | 50% to 100% increase |
| Poland | 417.7 | 677.0 | 62.1% | 50% to 100% increase |
| Portugal | 1704.8 | 3014.8 | 76.8% | 50% to 100% increase |
| Puerto Rico | 546.4 | 824.6 | 50.9% | 50% to 100% increase |
| Qatar | 45.6 | 755.4 | 1558.2% | >300% increase |
| Republic of Korea | 9115.5 | 20007.4 | 119.5% | 100% to 200% increase |
| Republic of Moldova | 216.5 | 196.7 | -9.1% | <30% decrease |
| Romania | 432.7 | 182.3 | -57.9% | 30% to 60% decrease |
| Russian Federation | 6402.1 | 6197.4 | -3.2% | <30% decrease |
| Rwanda | 522.6 | 1526.7 | 192.1% | 100% to 200% increase |
| Saint Kitts and Nevis | 8.1 | 15.4 | 88.7% | 50% to 100% increase |
| Saint Lucia | 36.2 | 59.6 | 64.9% | 50% to 100% increase |
| Saint Vincent and the Grenadines | 28.4 | 42.3 | 48.7% | <50% increase |
| Samoa | 96.7 | 298.3 | 208.6% | 200% to 300% increase |
| San Marino | 2.9 | 6.4 | 120.4% | 100% to 200% increase |
| Sao Tome and Principe | 16.9 | 97.0 | 474.0% | >300% increase |
| Saudi Arabia | 2377.9 | 15864.6 | 567.2% | >300% increase |
| Senegal | 1380.0 | 6684.2 | 384.3% | >300% increase |
| Serbia | 158.0 | 117.1 | -25.9% | <30% decrease |
| Seychelles | 7.6 | 27.4 | 259.9% | 200% to 300% increase |
| Sierra Leone | 474.2 | 3143.8 | 563.0% | >300% increase |
| Singapore | 614.9 | 1234.9 | 100.8% | 100% to 200% increase |
| Slovakia | 70.8 | 48.2 | -31.9% | 30% to 60% decrease |
| Slovenia | 28.7 | 19.7 | -31.5% | 30% to 60% decrease |
| Solomon Islands | 102.5 | 498.0 | 385.7% | >300% increase |
| Somalia | 622.3 | 3993.2 | 541.7% | >300% increase |
| South Africa | 5365.2 | 11457.3 | 113.5% | 100% to 200% increase |
| South Sudan | 437.8 | 1505.1 | 243.8% | 200% to 300% increase |
| Spain | 5755.3 | 10559.0 | 83.5% | 50% to 100% increase |
| Sri Lanka | 2003.1 | 5434.0 | 171.3% | 100% to 200% increase |
| Sudan | 2181.3 | 16246.3 | 644.8% | >300% increase |
| Suriname | 80.6 | 226.6 | 181.3% | 100% to 200% increase |
| Sweden | 917.9 | 1589.7 | 73.2% | 50% to 100% increase |
| Switzerland | 993.6 | 2220.3 | 123.5% | 100% to 200% increase |
| Syrian Arab Republic | 2033.7 | 5835.6 | 186.9% | 100% to 200% increase |
| Taiwan (Province of China) | 3742.4 | 4388.1 | 17.3% | <50% increase |
| Tajikistan | 381.1 | 1367.1 | 258.7% | 200% to 300% increase |
| Thailand | 5063.4 | 7957.0 | 57.1% | 50% to 100% increase |
| Timor-Leste | 45.8 | 293.9 | 541.5% | >300% increase |
| Togo | 331.2 | 1679.1 | 407.0% | >300% increase |
| Tokelau | 1.0 | 1.9 | 91.5% | 50% to 100% increase |
| Tonga | 45.1 | 100.7 | 123.2% | 100% to 200% increase |
| Trinidad and Tobago | 184.6 | 385.5 | 108.8% | 100% to 200% increase |
| Tunisia | 1030.9 | 3526.7 | 242.1% | 200% to 300% increase |
| Turkey | 4810.0 | 16915.5 | 251.7% | 200% to 300% increase |
| Turkmenistan | 234.9 | 877.7 | 273.7% | 200% to 300% increase |
| Tuvalu | 2.7 | 10.1 | 278.7% | 200% to 300% increase |
| Uganda | 1164.9 | 6813.7 | 484.9% | >300% increase |
| Ukraine | 2841.8 | 2356.9 | -17.1% | <30% decrease |
| United Arab Emirates | 145.1 | 1057.2 | 628.5% | >300% increase |
| United Kingdom | 7300.4 | 36222.5 | 396.2% | >300% increase |
| United Republic of Tanzania | 1507.2 | 7218.9 | 378.9% | >300% increase |
| United States of America | 11425.2 | 38468.8 | 236.7% | 200% to 300% increase |
| United States Virgin Islands | 19.0 | 19.9 | 4.7% | <50% increase |
| Uruguay | 102.9 | 324.6 | 215.4% | 200% to 300% increase |
| Uzbekistan | 1638.3 | 5232.7 | 219.4% | 200% to 300% increase |
| Vanuatu | 48.6 | 291.5 | 499.9% | >300% increase |
| Venezuela (Bolivarian Republic of Venezuela) | 3075.3 | 6204.1 | 101.7% | 100% to 200% increase |
| Viet Nam | 4324.2 | 8753.5 | 102.4% | 100% to 200% increase |
| Yemen | 1068.9 | 8311.6 | 677.6% | >300% increase |
| Zambia | 1107.9 | 6669.6 | 502.0% | >300% increase |
| Zimbabwe | 969.9 | 2844.8 | 193.3% | 100% to 200% increase |
